# Clinicopathologic Analysis of Dermatofibroma: A Retrospective Study of 165 Cases

**DOI:** 10.7759/cureus.82305

**Published:** 2025-04-15

**Authors:** Jiao He, Hui Chen, Zhi Duan, Hua He, Ting Tao

**Affiliations:** 1 Department of Pathology, The First Hospital of Changsha, Changsha, CHN

**Keywords:** clinicopathologic analysis, dermatofibroma, gender, horizontal tumor size, retrospective study

## Abstract

Dermatofibroma (DF), also known as benign fibrous histiocytoma, is a common benign skin tumor whose clinicopathologic features and pathogenesis remain only partially understood. In this retrospective study, 165 cases of DF diagnosed between 2018 and 2024 were analyzed to characterize demographic, clinical, and histopathologic profiles. Data regarding patient age, gender, horizontal tumor size, anatomical location, and pathological subtypes were extracted from digital pathology archives. Statistical analyses revealed that male patients exhibited significantly larger horizontal tumor sizes compared to female patients (P = 0.027). Additionally, the tumor location was significantly associated with size, with lesions in the subcutaneous tissue showing larger mean horizontal dimensions than those in the reticular or papillary dermis (P = 0.032). These findings suggest that gender, tumor location, and pathological subtype are influential factors in DF growth, providing further insight into its clinical behavior and potential underlying mechanisms. These findings highlight the importance of considering patient gender and tumor location in the clinical management of DF, potentially guiding personalized treatment strategies and improving patient outcomes.

## Introduction

Dermatofibroma (DF), also known as benign fibrous histiocytoma, is one of the most common benign tumors in dermatology, accounting for approximately 3% of all pathological specimens [1]. The condition affects a broad population, with a higher incidence observed in young to middle-aged women [2]. Clinically, DF typically presents as a solitary, firm nodule with a reddish or brownish surface and is often accompanied by a characteristic “dimple sign” upon lateral compression. The lesions usually measure less than 1 cm in diameter, and several cohort studies have reported a predilection for the lower extremities, with surgical excision generally resulting in a favorable prognosis [3,4].

DF consists of fibroblasts and fibrous tissue cells within the dermis, exhibiting a classic histopathological appearance characterized by spindle cells arranged in a storiform pattern with dense bundles of collagen fibers, and in some cases, overlying epidermal hyperplasia. DF encompasses several histopathologic subtypes, including aneurysmal DF [5], lipidized DF [4], atypical DF [6], atrophic DF [7], and cellular DF [8]. These diverse morphological variants can pose diagnostic challenges, particularly when differentiating DF from other soft tissue neoplasms. The precise pathogenesis of DF remains unclear. Some studies suggest that DF may represent a true neoplasm, as evidenced by consistent ALK expression and ALK gene rearrangements in epithelioid DF, as well as gene fusions involving PRKCB and PRKCD, which have been detected across various DF subtypes. Notably, certain subtypes, such as aneurysmal DF and cellular DF, have been associated with recurrence and, in rare cases, metastasis [9-11]. Conversely, other reports have linked DF development to tissue injury, such as insect bites or folliculitis, supporting the hypothesis that DF may arise as a reactive process following local trauma [12]. Therefore, despite its frequent occurrence in clinical practice, the exact pathogenesis and clinicopathologic characteristics of DF across different populations remain incompletely understood.

In this study, we retrospectively analyzed 165 cases of DF diagnosed by our pathology department from 2018 to 2024. Although large cohort studies of DF are abundant, our study is distinctive in that it represents a six-year retrospective analysis from a regional hospital. Importantly, our findings reveal two novel aspects: first, we identified a significant gender-related difference in horizontal tumor size with male patients exhibiting larger tumor diameters than females, a finding that has not been previously reported in the literature; and second, we found that the cellular subtype of DF exhibits a significantly larger horizontal tumor size compared to the classic subtype. These unique observations provide further insight into the clinicopathologic spectrum of DF and underscore the potential influence of both gender and histologic subtype on tumor behavior.

## Materials and methods

Study design and data source

This study was a retrospective analysis of pathological data from patients diagnosed with DF. A total of 165 cases were retrieved from the digital pathology archive stored in our local database, spanning from 2018 to 2024. These digital slides, generated from surgical specimens, were preserved for histopathological evaluation. To protect patient confidentiality, all patient information was de-identified and anonymized prior to analysis. The study aimed to characterize the epidemiological and pathological features of DF based on available clinical and histological data.

Inclusion and exclusion criteria

Cases were included if they had a histopathologically confirmed diagnosis of DF, available basic demographic and clinical information (such as sex, age, horizontal tumor size, and tumor location), and underwent surgical excision with corresponding digital pathology slides archived in the database. Cases were excluded if key information, such as horizontal tumor size or tumor location, was missing, rendering them unsuitable for epidemiological analysis. Of the initial 174 cases, nine cases were excluded due to incomplete data, leaving 165 cases for the final statistical analysis.

Data collection

Data were extracted from pathology reports linked to each digital slide. Recorded variables included gender, age, horizontal tumor size (measured in centimeters as the maximum dimension along the tumor’s long axis from the largest surgical specimen cross-section), tumor location (anatomical site), diagnostic classification (histological subtypes: classic, aneurysmal, hemosiderotic, cellular, lipidized, or atrophic), and pathological features such as tumor center (papillary dermis, reticular dermis, or subcutaneous tissue), epidermal changes (hyperkeratosis, acanthosis), keloidal collagen, foam cells, giant cells, hemosiderin deposition, and special changes (e.g., epidermal ulceration). Missing data were noted, and only cases with complete records for sex, age, horizontal tumor size, and tumor location were included in the epidemiological analysis. All specimens underwent surgical excision, with tumor's horizontal diameter measured post-excision and reviewed by two senior dermatopathologists to ensure diagnostic accuracy.

Ethics approval

This study received ethical approval from the First Hospital of Changsha under the reference number "(2024) Ethics Fast-track Review [Clinical Research] No. (63)."

Statistical analysis

Data analysis was performed using R software (version 4.4.2; R Foundation for Statistical Computing, Vienna, Austria). Descriptive statistics, including means, standard deviations, frequencies, and percentages, were calculated for continuous variables (e.g., age, horizontal tumor size) and categorical variables (e.g., sex, tumor location). Normality was assessed with the Shapiro-Wilk test, and variance homogeneity was evaluated using Levene’s and Bartlett’s tests. For comparative analysis, independent sample t-tests compared means of continuous variables between two groups (e.g., sex) when normality was met, while the Mann-Whitney U test was used when normality was violated; one-way ANOVA compared means across multiple groups (e.g., age groups) with normality and homogeneity assumed, otherwise the Kruskal-Wallis test was applied; chi-square and Fisher’s exact tests analyzed associations between categorical variables (e.g., diagnostic classification and pathological features), with Fisher’s test used for low expected frequencies. Spearman’s rank correlation assessed monotonic relationships between continuous variables (e.g., horizontal tumor size and age). Statistical significance was set at p < 0.05, and incomplete fields beyond core variables were excluded from sub-analyses to maintain data integrity. Digital slides were securely stored in the local database.

## Results

**Figure 1:**
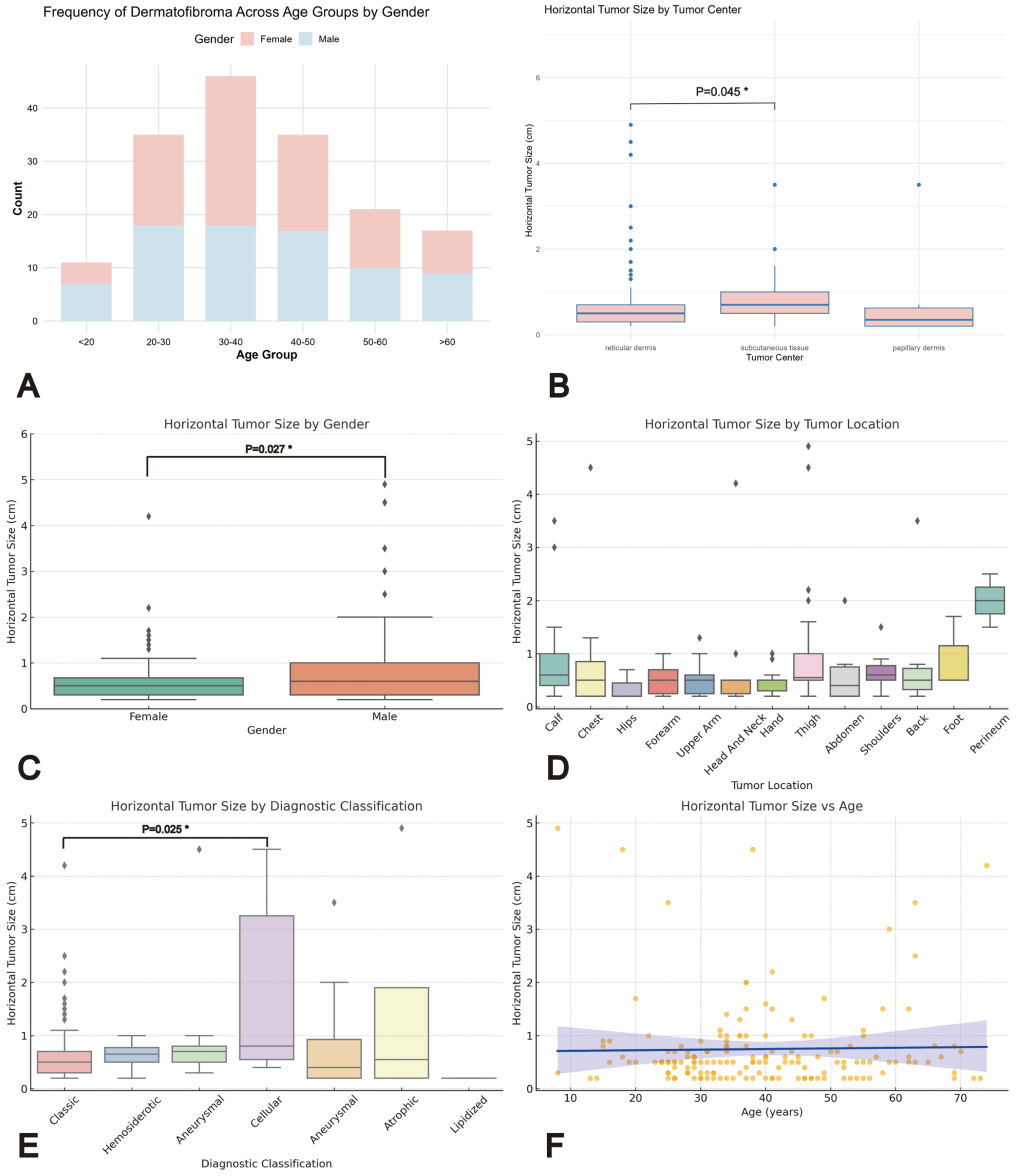
A: Frequency of dermatofibroma across age groups by gender (stacked column chart). B: Horizontal tumor size by tumor center (boxplot). C: Horizontal tumor size by gender (boxplot). D: Horizontal tumor size by tumor location (boxplot). E: Horizontal tumor size by diagnostic classification (boxplot). F: Scatter plot of horizontal tumor size vs age (linear regression). Most DFs were in the reticular dermis (72.7%), followed by the subcutaneous tissue (21.8%) and papillary dermis (5.5%). Tumors in the subcutaneous tissue had the largest mean horizontal size (0.85 cm) compared to those in the reticular (0.72 cm) and papillary (0.76 cm) dermis. Statistical analysis confirmed significant differences (Kruskal-Wallis, P = 0.032; Wilcoxon, reticular vs. subcutaneous, P = 0.045). *A P-value less than 0.05 is generally considered statistically significant. DF: Dermatofibroma

Figure 1 comprehensively illustrates the clinical and pathological characteristics of the studied DF cases. Figure 1A shows the age distribution of the patient cohort, while Figure 1B demonstrates the significant influence of tumor center location on horizontal tumor size. Figure 1C compares the horizontal tumor size between male and female patients, and Figure 1D further breaks down the horizontal tumor size across different anatomical locations, highlighting gender-specific differences in the lower extremities. Figure 1E displays the variation in horizontal tumor size among different DF subtypes, and finally, Figure 1F presents the linear regression analysis exploring the relationship between horizontal tumor size and patient age.

Clinical characteristics

A total of 165 cases with histopathologically confirmed DF were included in this study. The patients' ages ranged from 8 to 74 years, with a mean age of 39.11 ± 14.31 years. The distribution of cases across different age groups is illustrated in Figure 1A. Regarding gender, female patients accounted for 86 cases (52.1%), while male patients comprised 79 cases (47.9%), indicating a slightly higher proportion of female patients.

The horizontal tumor size ranged from 0.20 to 4.90 cm, with a mean size of 0.75 ± 0.82 cm; the median size was 0.5 cm in female patients and 0.6 cm in male patients. Statistical analysis using the Mann-Whitney U test revealed that the horizontal tumor size was significantly larger in male patients than in female patients (P = 0.027, Figure 1C). The most common sites of tumor occurrence were the thigh (26 cases), calf (21 cases), upper arm (19 cases), forearm (15 cases), and hand (13 cases). Although there were no statistically significant differences in the horizontal tumor size between different anatomical locations, male patients had larger tumor sizes in the lower extremities (Table 1 and Figure 1D).

**Table 1:** Epidemiological and pathological characteristics of dermatofibroma (DF) patients. Note: Horizontal tumor size data are expressed as “mean ± standard deviation”; percentages are calculated based on the total sample size (n=165); tumor location and diagnostic classification (such as lipidized) with a frequency lower than 5% are not listed in the table.

Group	Classification	N (%)	Horizontal Tumor Size (cm)	P-Value
Gender	Not Available			0.027
	Female	86 (52.12)	0.60 ± 0.55	
	Male	79 (47.88)	0.91 ± 1.01	
Age (Years)	Not Available			0.507
	<20	11 (6.67)	1.30 ± 1.70	
	20-39	81 (49.10)	0.68 ± 0.67	
	40-59	56 (33.90)	0.64 ± 0.55	
	≥60	17 (10.30)	1.06 ± 1.19	
Tumor Locations	Not Available			0.133
	Thigh	26 (15.80)	1.03 ± 1.20	
	Calf	21 (12.70)	0.93 ± 0.88	
	Upper Arm	19 (11.50)	0.49 ± 0.29	
	Forearm	15 (9.09)	0.51 ± 0.29	
	Chest	12 (7.27)	0.87 ± 1.20	
Diagnostic Classification	Not Available			0.208
	Classic	128 (77.60)	0.64 ± 0.54	
	Aneurysmal	21 (12.70)	0.92 ± 1.12	
	Hemosiderotic	4 (2.42)	0.62 ± 0.33	
	Cellular	7 (4.24)	1.90 ± 1.71	
	Atrophic	4 (2.42)	1.55 ± 2.26	
Tumor Center	Not Available			0.032
	Papillary Dermis	8	0.76 ± 1.12	
	Reticular Dermis	129	0.72 ± 0.83	
	Subcutaneous Tissue	28	0.85 ± 0.83	

Pathological features

The horizontal tumor size varied significantly among different subtypes of DF. The mean tumor sizes for the five major subtypes were as follows: classic DF (0.64 ± 0.54 cm), cellular DF (1.90 ± 1.71 cm), atrophic DF (1.55 ± 2.26 cm), aneurysmal DF (0.83 ± 0.99 cm), and hemosiderotic DF (0.63 ± 0.33 cm). Notably, as shown in Figure 1E, the cellular subtype demonstrated a significantly larger horizontal tumor size compared to the classic subtype (Mann-Whitney U test, P = 0.025).

Tumor center also influenced the horizontal tumor size significantly (Figure 1B). Most DFs were located in the reticular dermis (72.7%), followed by the subcutaneous tissue (21.8%) and papillary dermis (5.5%). The mean horizontal tumor size for tumors located in the subcutaneous tissue was greater (mean size: 0.85 cm) than those located in the reticular dermis (mean size: 0.72 cm) or papillary dermis (mean size: 0.76 cm). A Kruskal-Wallis test confirmed these differences as statistically significant (P = 0.032). Further pairwise comparisons using the Wilcoxon test revealed a significant difference between tumors located in the reticular dermis and subcutaneous tissue, with larger sizes observed in the subcutaneous tissue group (P = 0.045).

Epithelial and specific lesions

Epithelial lesions such as hyperkeratosis and acanthosis were observed in a subset of cases, with hyperkeratosis present in 29 cases (17.6%) and acanthosis noted in only one case (0.6%). Comparison of horizontal tumor sizes between cases with and without epithelial lesions showed no statistically significant differences; tumors with hyperkeratosis had a mean size of 0.73 ± 0.62 cm, while tumors without epidermal changes had a mean size of 0.79 ± 0.84 cm (Mann-Whitney U test, P > 0.05).

Specific pathological features, including keloidal collagen, foam cells, giant cells, hemosiderin deposition, and epidermal ulcers, were observed sporadically among individual cases but did not show statistically significant differences in tumor size between groups due to limited sample sizes. Interestingly, chronic skin inflammation was associated with markedly larger tumor diameters (mean size: 3.5 cm), while epidermal ulcers corresponded to smaller tumor diameters (mean size: 0.65 cm). However, statistical significance could not be established due to small sample sizes within these subsets.

Microscopic features

**Figure 2:**
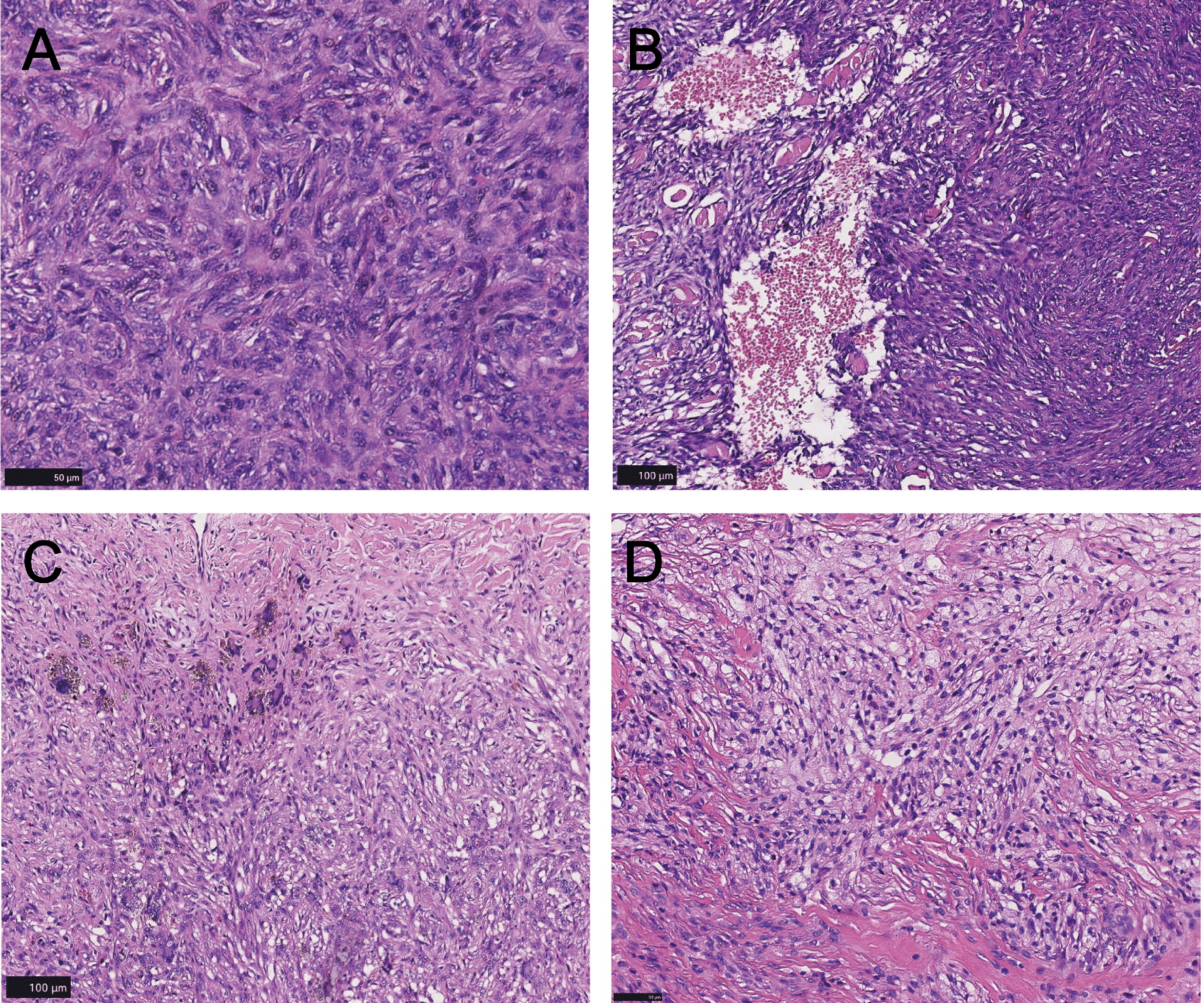
Microscopic features of dermatofibroma. A: The tumor is mainly composed of spindle cells, which are interwoven and densely packed (scale bar = 50 µm). B: Aneurysmal fibrous histiocytoma shows typical hyperemic cystic components (scale bar = 100 µm). C: Hemosiderin deposition can be seen in the tumor stroma, accompanied by multinucleated giant cells arranged in a wreath shape (scale bar = 100 µm). D: The cytoplasm of the tumor cells has a foamy appearance (scale bar = 50 µm).

In our study cohort, classic DF emerged as the most common subtype. The lesions were predominantly located within the dermis and presented as solitary, small nodules with well-defined borders. In a minority of cases, tumor cells infiltrated the surrounding adipose tissue. Microscopically (Figure 2), the tumor cells were primarily spindle-shaped and interwoven, often accompanied by areas of collagenization. Occasionally, foamy histiocytes were observed, and multinucleated giant cells appeared sporadically. Hemosiderin deposition was noted in some tumors, likely due to trauma or hemorrhage. The tumor cells were small, with abundant cytoplasm and oval or elliptical nuclei, exhibiting a nuclear-to-cytoplasmic ratio of 1:1 or 1:2. Mild nuclear atypia was present, with mitotic figures generally fewer than 5 per 20 high-power fields. Collagen fibers, a hallmark of this condition, were arranged in curved or reticular patterns. Fibrosis was observed in the stroma of certain tumors, with collagen fibers displaying irregular or bundled arrangements.

Among our cohort, 21 patients were diagnosed with aneurysmal DF. Histologically, this subtype was characterized by fibrohistiocytic proliferation surrounding blood-filled spaces lacking vascular endothelial lining, forming distinctive cystic and luminal structures. Thrombosis was occasionally identified within these spaces. As the disease progressed, hemosiderin deposition became more prominent, complicating differential diagnosis.

## Discussion

DF is one of the soft tissue tumors of the skin, typically occurring in middle-aged individuals, although it can affect people of all ages, with a slightly higher incidence in women than in men. These tumors generally present as solitary lesions, most often oval or round in shape, with a relatively firm texture, and almost always arise in the reticular dermis [4]. In our retrospective analysis, we observed a slightly higher number of female patients, and the median age of the patients was 37 years. Based on the distribution of tumor occurrence at various anatomical sites, the lower extremities, particularly the thighs and calves, exhibited a higher incidence than the upper extremities, a finding that is highly consistent with previous reports [3,13]. Although most patients did not report a history of significant injury, minor traumas such as insect bites and friction may still act as triggers for tumor formation [14]. Furthermore, the unique physiological environment of the lower extremities, for example, wearing tight clothing or engaging in frequent physical activity, may result in persistent mechanical pressure on the local tissues, leading to micro-injuries and subsequent excessive proliferation of fibrous tissue.

Notably, we found that the horizontal tumor size in male patients was significantly larger than that in female patients. This suggests that the horizontal diameter of DF may be influenced by gender, implying that gender factors might play a role in the growth patterns or developmental mechanisms of these tumors. Previous studies have indicated that sex hormones play an important role in regulating disease progression, with estrogen believed to exert a protective effect against tissue fibrosis [15]. Given that estrogen and progesterone levels are higher in women [16], these hormones may modulate the proliferation and repair mechanisms of the skin, thereby inhibiting the formation and growth of DF. In contrast, the relatively higher levels of testosterone in men may be closely associated with increased tumor size [17], warranting further investigation into its underlying mechanisms and clinical significance.

In terms of pathological classification, the cellular subtype exhibited a significantly larger tumor size, suggesting that cellular DF may have distinct biological behavior or clinical prognosis compared to the classic subtype [8]. Although other subtypes, such as atrophic, aneurysmal, and hemosiderotic ones, showed some differences in tumor size, no statistically significant differences were observed in our cohort. We speculate that this may be due to the limited number of tumors in these subtypes, resulting in an insufficient sample size.

Analysis based on the tumor location indicates that most DFs are located in the reticular dermis, followed by the subcutaneous tissue and papillary dermis, with the horizontal tumor size of lesions in the reticular dermis being smaller than those in the subcutaneous tissue. The reticular dermis, as the deeper layer of the dermis, lies beneath the papillary dermis and constitutes the main component of the dermis. In contrast, the subcutaneous tissue, the innermost layer located beneath the dermis, is primarily composed of adipose tissue, with fat cells clustered within lobules separated by connective tissue. The dense collagen fibers in the reticular dermis may offer greater resistance to tumor growth, acting like a constraining scaffold, whereas the less dense structure of the subcutaneous tissue may provide less resistance. Moreover, the richer vascular network in the subcutaneous tissue could supply developing tumors with more nutrients and growth factors, while the microenvironment of the small DFs confined to the reticular dermis may be less vascularized. Consequently, the high density of collagen and limited blood supply in the reticular dermis restrict tumor growth [18]. Additionally, the tumor microenvironment (TME) may play a crucial role in promoting tumor growth; the TME in the subcutaneous tissue is primarily composed of adipocytes, connective tissue, larger blood vessels, and nerves. Adipocytes are known to engage in complex interactions with cancer cells and facilitate tumor progression, as demonstrated in many in vitro experiments [19]. By secreting adipokines, cytokines, or growth hormones [20,21], adipocytes constitute a major component of the TME that supports cancer growth. Some studies have indicated that isolated adipocytes supply palmitic acid, which promotes the growth of melanoma cells by activating Akt in a PTEN-independent manner [22]. This raises the hypothesis that adipocytes might similarly influence the growth of DF.

Although the WHO classifies DF as a type of skin tumor, some scholars argue that DF is not a true neoplasm but rather a result of local trauma, inflammation, and repair responses. According to previous studies, the development of DF is typically divided into three phases: the granulomatous phase, the inflammatory phase, and the fibrotic phase. The initial stage is characterized by a massive infiltration of histiocytes, which gradually transitions to fibroblast proliferation and ultimately forms a distinct tumor nodule [23]. In this process, the tumor gradually shifts from an early immune response phase to a proliferation phase dominated by fibroblasts, which may be closely related to the tissue repair requirements. Other studies have suggested that DF may be a lesion with heterogeneous origin, proposing that it results from a fusion of reactive fibroblast proliferation and neoplastic components. This view holds that the fibrous tissue component in DF might not be entirely neoplastic but rather a reactive proliferation in response to local trauma or inflammation, with the proliferation of fibroblasts being closely related to the body's repair mechanisms, while the proliferation of histiocyte-like cells may represent true neoplastic growth [24]. Furthermore, some studies using X-chromosome inactivation analysis have found clonal markers in DF cells, indicating monoclonality, which may support a neoplastic origin for DF [25,26]. This suggests that the pathogenesis of DF may be more complex than a simple tumor proliferation, warranting further investigation.

The main limitations of this study include its retrospective design and the fact that the data were derived from a single institution over the past five years, which may limit the generalizability of the findings. Additionally, not all rare subtypes of DF were comprehensively included, which may limit the study's ability to capture the full diversity and complexity of DF. Furthermore, the lack of dermoscopic images and corresponding clinical diagnoses prevented us from assessing the accuracy of clinical diagnoses. Another limitation is the absence of clinical follow-up data, such as recurrence rates and patient outcomes, which hindered our ability to evaluate the long-term progression and prognosis of dermatofibromas.

Our comprehensive analysis highlights the importance of considering gender and tumor subtype in the management of dermatofibromas, revealing that gender is an important factor influencing DF size, with male patients tending to develop larger tumors and potentially requiring more proactive monitoring. Additionally, the cellular subtype, characterized by its larger size, may necessitate closer surveillance or more aggressive treatment compared to the classic subtype. The high incidence of DF in the lower extremities suggests that patients with repetitive mechanical stress or minor trauma may be at higher risk, warranting consideration of preventive measures. Furthermore, tumor anatomical location, particularly its depth, may influence its growth potential, and the TME, especially adipocytes within subcutaneous tissue, may play a key role in promoting DF growth, potentially informing future therapeutic strategies aimed at limiting DF growth. 

## Conclusions

Our retrospective analysis of 165 cases of DF reveals that gender is a significant factor influencing tumor size, with male patients tending to develop larger lesions. Tumor center also plays a critical role, as DF in the subcutaneous tissue exhibits larger horizontal dimensions compared to those in the reticular or papillary dermis. Moreover, the cellular subtype shows a more aggressive profile with a significantly larger tumor size relative to the classic subtype. These findings suggest that biological factors such as sex hormones and the TME, particularly the role of adipocytes, may be pivotal in DF development and progression. Although the study is limited by its retrospective design and single-institution data, it provides valuable insights into the clinicopathologic characteristics of DF and lays the groundwork for future large-scale investigations into its underlying mechanisms.
